# R-Loops and Its Chro-Mates: The Strange Case of Dr. Jekyll and Mr. Hyde

**DOI:** 10.3390/ijms22168850

**Published:** 2021-08-17

**Authors:** Sidrit Uruci, Calvin Shun Yu Lo, David Wheeler, Nitika Taneja

**Affiliations:** 1 Department of Molecular Genetics, Erasmus MC Cancer Institute, Erasmus University Medical Center, 3000 CA Rotterdam, The Netherlands; s.uruci@erasmusmc.nl (S.U.); s.lo@erasmusmc.nl (C.S.Y.L.); 2 Laboratory of Biochemistry and Molecular Biology, National Cancer Institute, NIH, Bethesda, MD 20892, USA; dwheeler@helix.nih.gov

**Keywords:** R-loops, RNA:DNA hybrids, histone modifiers, chromatin remodelers, R-T conflicts, genome stability, DNA damage

## Abstract

Since their discovery, R-loops have been associated with both physiological and pathological functions that are conserved across species. R-loops are a source of replication stress and genome instability, as seen in neurodegenerative disorders and cancer. In response, cells have evolved pathways to prevent R-loop accumulation as well as to resolve them. A growing body of evidence correlates R-loop accumulation with changes in the epigenetic landscape. However, the role of chromatin modification and remodeling in R-loops homeostasis remains unclear. This review covers various mechanisms precluding R-loop accumulation and highlights the role of chromatin modifiers and remodelers in facilitating timely R-loop resolution. We also discuss the enigmatic role of RNA:DNA hybrids in facilitating DNA repair, epigenetic landscape and the potential role of replication fork preservation pathways, active fork stability and stalled fork protection pathways, in avoiding replication-transcription conflicts. Finally, we discuss the potential role of several Chro-Mates (chromatin modifiers and remodelers) in the likely differentiation between persistent/detrimental R-loops and transient/benign R-loops that assist in various physiological processes relevant for therapeutic interventions.

## 1. Introduction

**Figure 1:**
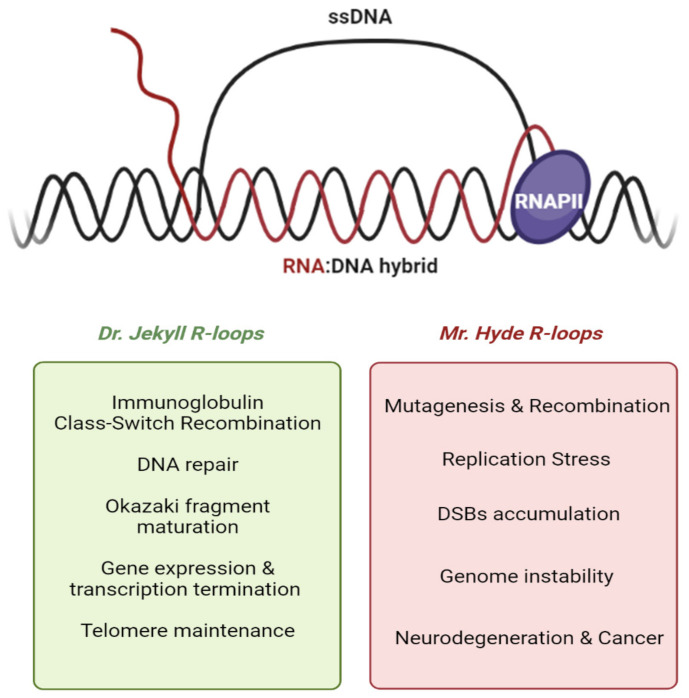
(**Top**) Schematics of R-loop structure showing RNA:DNA hybrid formed between the nascent RNA (in red) and the transcribed DNA template, while a non-transcribed template is displaced as ssDNA. (**Bottom**) In the green box are listed the main and most studied implications of R-loops in physiological contexts. In the red box, conversely, are listed the most noteworthy roles of persistent and detrimental R-loops.

The unwinding of the DNA double helix during events such as transcription, DNA replication or DNA repair, offers the opportunity for various anomalies, such as RNA:DNA hybrids or R-loops to form. R-loops were first discovered more than 40 years ago [1] and were named as “R-loops” to depict the three-stranded structure similar to previously described D-loops [2], but with an RNA moiety in the hybrid. It is still not fully understood how R-loops are generated. The most popular model, the “thread back model”, is supported by crystallographic analysis [3,4] and suggests that the newly formed RNA strand threads back between the two DNA strands before they reanneal, forming the RNA:DNA hybrid and displacing the non-template single-strand DNA [5,6]. Initially, R-loops were thought to be transient and to be produced only as a byproduct of transcription, but their greater significance is now coming to light [7,8,9,10]. Although it is unclear how R-loops are generated, more is known about the regions in which they form and accumulate. R-loops are usually prone to form whenever the DNA template is C-rich, annealing with a G-rich RNA transcript [11]. In particular, R-loops originate at regions called R-loop Initiation Zones (RIZ) containing few clusters of Gs. Downstream of these regions, R-loops elongate in the R-loop Elongation Zones (REZ) that contain a higher proportion of G residues. It is this difference in G density between RIZ and REZ regions that permits the strand unwinding required for R-loop formation [5]. The process of R-loop formation is thermodynamically favored since the RNA:DNA hybrid interaction is much stronger and more stable than that of DNA:DNA duplexes [12,13]. There are many theories as to why this may be the case and, in addition to GC skew (the asymmetric distribution of G and C residues along the two strands), negative supercoils and DNA nicks are believed to play a fundamental role in RNA:DNA interaction and thus in enhancing R-loop stability. For instance, negative supercoils promote the separation of two strands of DNA, providing a chance for RNA to thread back and anneal with the DNA template. DNA nicks on the non-template ssDNA tend to lower the binding affinity of the two DNA strands, making it harder for them to reanneal, also promoting RNA:DNA hybrid interaction [14]. It is important to highlight that R-loop formation is not a rare event as was previously thought. Deep-sequencing techniques have revealed that about 60% of the human transcribed genome contains at least one R-loop Forming Sequence (RLFS) [15]. Furthermore, R-loops have been observed in the genomes of organisms ranging from bacteria [16,17] to yeast [18,19], plants [20,21] and mammals [22,23]. In human, R-loops are formed at a modest frequency that ranges from 0.5% to 10% along tens of thousands of highly conserved hotspots [23,24]. R-loops’ roles are often divided into two main categories: “scheduled” R-loops, which are involved in normal physiological pathways of the cell cycle, and “unscheduled” R-loops, that appear to form only during episodes of cellular dysregulation and have been linked to replication stress, DNA damage, and to several human pathologies such as neurodegenerative diseases and cancer [8,10] (Figure 1). RNA:DNA hybrids can thus be considered as a double-edged sword, especially in the processes of DNA replication and transcription where their pathological roles are a source of replication stress and lead to genome instability [6].

## 2. Dual Nature of R-Loops

RNA:DNA hybrids are a natural intermediate of both DNA replication and mRNA synthesis. During DNA replication, an RNA:DNA hybrid of 18–22 bp is formed to begin priming by DNA polymerase α and is later used by DNA polymerase δ to synthesize each Okazaki fragment on the lagging strand. These structures have a transient nature since they are ultimately removed by nucleases such as RNase H enzymes [25,26]. Small RNA:DNA hybrids also form whenever DNA polymerase ε misincorporates ribonucleotide monophosphates (rNMPs) during leading strand synthesis. These misincorporated nucleotides can play a detrimental role if not properly processed. However, rNMPs incorporation also serves an important role in the signaling of DNA damage and in directing the repair apparatus of the Mismatch Repair (MMR) and Non-Homologous End-Joining (NHEJ) pathways [27,28,29]. A transient RNA:DNA hybrid of 8–9 bp is also produced within the transcription bubble by RNA polymerases as a step in RNA synthesis [4,30]. In this process, RNA is briefly linked with the template DNA strand; however, the newly synthesized transcript and the DNA template are extruded from different exit channels of the RNA polymerase II (RNAPII), thus normally preventing hybrid extension outside the transcription bubble [4,30,31].

Formation and stabilization of R-loops is involved in physiological processes, most notably the Class-Switch Recombination (CSR) at the Immunoglobulin (Ig) heavy chain locus in B lymphocytes, through which antibody isotypes are generated [32]. CSR occurs at a specific locus, a repetitive switch region located downstream of a promoter that tends to be longer than 1 Kbp with repeat units of 25–80 bp [32]. The displaced ssDNA of the R-loop structure is targeted by the Activation-Induced Deaminase (AID) that deaminates cytosine to produce uracil [33]. These new uracil residues are removed using DNA repair pathways such as Base Excision Repair (BER) and MMR, leading to a scheduled double-strand break (DSB) accumulation and a high recombination rate in the switch region which allows CSR at the IgH genes [34]. On the other hand, uncontrolled AID activity can create pathological DNA nicks that are resolved by the error-prone BER pathway, leading to spontaneous DSB accumulation that poses a natural barrier for fork progression [33,34,35]. Moreover, these mutations can also lead to chromosomal translocation as seen in the strong *IgH* enhancers which translocate close to the *c-Myc* promoter, resulting in its over-expression in Burkitt lymphoma [36].

For a time, CSR was believed to be the only normal physiological role in which R-loops are involved, but several studies have highlighted their involvement in chromatin modification and consequently in the regulation of gene expression. The role of R-loops in the regulation of gene expression is evident by its role in transcription termination. The R-loops formation at the 3′-end of the gene causes the pausing of RNAPII downstream of the poly(A) signal to promote termination [22]. These R-loops are resolved by the RNA:DNA helicase Senataxin (SETX) followed by degradation mediated by the 5′-3′ exoribonuclease 2 (Xrn2) nuclease [37]. R-loops are also formed at gene promoters and adjacent CpG islands. CpG islands are composed of CpG dinucleotides with a high GC skew that favors R-loop formation. R-loops within CpG islands preclude DNA methylation of the nearby promoter region, allowing subsequent gene expression [11].

Interestingly, the co-occurrence of R-loops in association with epigenetic marks such as DNA methylation and certain histone modifications (discussed in Section 7) has also been proposed to have a role in regulating the chromatin landscape [23,38,39]. Whether the R-loops are the consequence of chromatin landscape modulation or whether transiently formed R-loops serve as an additional epigenetic layer sensed by chromatin modifying and remodeling enzymes to induce changes in chromatin state is an interesting question.

It is not the R-loop formation per se that hinders genome stability, since, as discussed above, their transient and scheduled formation is required for the physiological processes that they regulate. Rather, it is the unscheduled and uncontrolled accumulation of persistent R-loops that leads to DNA damage and genome instability. R-loop structure alone has a destabilizing effect on genome stability as the unstable displaced ssDNA is highly susceptible to nucleases and genotoxins, resulting in transcription-associated mutagenesis (TAM) and transcription-associated recombination (TAR). This, in turn, leads to the accumulation of detrimental DSBs causing DNA damage [7,8,40,41]. Additionally, the formation of another non-canonical secondary DNA structure, the G-quadruplex (G4), has been demonstrated along the ssDNA within R-loops [42]. The guanines in G4 structures interact through a Hoogsteen hydrogen bond stabilized by a cation and form a planar conformation of guanine stacks stabilized by π-π bond interaction [43]. G4s, as well as R-loops, are implicated in normal physiological roles as they are both associated with replication origins [44]. G4s and R-loops are structurally compatible since both tend to form within G-rich, negatively supercoiled sequences [45]. The formation of G4s within the ssDNA of R-loops, a structure called the G-loop [42] stabilizes R-loops by extending the RNA:DNA hybrid length [45]. G4s and R-loop accumulation during transcription constitute a coupled mechanism with consequences for genome and epigenome instability [46,47], however, the reason for their strong co-occurrence is still unclear.

## 3. R-Loops Accumulation: Why and Where

Unscheduled and persistent R-loops can endanger genome stability primarily due to their ability to perturb replication fork progression and by generating DNA breaks. R-loops are the most detrimental outcome of replication-transcription (R-T) conflicts as they share the same DNA template during the S-phase of the cell cycle, making conflict an inevitable event [48,49,50,51,52,53,54]. Since both replication and transcription possess a 5′-to-3′ polarity, the conflict between these machineries can occur in two different fashions, based on the gene orientation; a head-on (HO) collision in the lagging strand or a co-directional (CD) collision in the leading strand [50,55]. The difference between the two dynamics is yet to be elucidated, however, the results of recent studies suggest that HO conflicts exacerbate R-loop stabilization and accumulation [48,56,57,58]. Thus, the conflict orientation might determine whether R-loops accumulate with stabilization being favored during HO conflicts and conversely being disfavored during CD conflicts. The latter CD conflicts are likely to be less detrimental to genome stability, and may even have a role in the resolution of replication stress [55,57]. During HO conflicts, RNAPII acts as an impediment to fork progression, leading to fork stalling at transcribed units as revealed by two-dimensional gel electrophoresis [57,59,60,61]. It was observed that high levels of R-loops are preferentially associated with HO conflicts [16,55,58]. However, the dynamics of R-loops formation at the site of HO conflicts are still unclear. Therefore, whether HO conflicts, resulting in slow moving or stalled replication forks, are caused by the accumulation of R-loops or rather these conflicts are the reason for R-loops accumulation behind the trapped RNAPII machinery, is still elusive [43,48,49,62].

The R-T conflicts are more prominent in difficult-to-replicate genomic regions, including fragile sites, that are generally rich in repetitive sequences and/or are long expressed genes. The fragile sites are more prone to accumulate replication stress leading to chromosomal gaps and breaks and thus to gross chromosomal rearrangements, a well-known hallmark of cancer [63]. Such regions have also been associated with trinucleotide repeat expansion syndromes, such as Fragile X Syndrome [64] that causes replication fork stalling and play a role in the onset of genome instability. There are two main classes of fragile sites, based on their inheritance pattern: Rare Fragile Sites (RFSs) and Common Fragile Sites (CFSs). RFSs are present in less than 5% of the population and are usually associated with rare Mendelian inherited traits through the expansion of trinucleotide repeats such as the CGG triplet of FRAXA, a fragile site on the *FMR1* gene associated with the Fragile X Syndrome [65,66,67]. RFSs are sites that are replicated late since the trinucleotide expansion makes them more prone to accumulate secondary structures that efficiently block fork progression [68]. CFSs are commonly associated with long genes (>800 Kbp), which may be the consequence of inevitable conflicts between replication and transcription machineries at these sites [69]. CFSs are also identified in late-replicating regions that are intrinsically unstable. With CFSs there is a paucity of active replication origins and of dormant origins, implying that, once fired, the forks in this region have to travel long distances without the possibility of rescue from benign mechanisms, such as dormant origin firing. Low origin density exacerbates the effects of collisions that may occur in these regions, causing replication stress, R-loop accumulation and incomplete DNA synthesis [70,71,72,73]. Cells entering mitosis bearing under-replicated DNA at these loci have been proven to have a defective chromatin compaction during anaphase. The defective compaction of such regions in mitotic chromosomes results in the formation of ultra-fine anaphase bridges (UFBs), a physical link between homologous chromosome that impedes correct segregation of chromosomes, causing breaks and leading to gross chromosomal aberrations [43,74,75]. Apart from these two classes of fragile sites, a new category of fragile sites has also been identified, namely, the Early Replicating Fragile Sites (ERFSs) [76]. Contrary to CFSs, these regions are early-replicating and are located in close proximity to replication origins and to highly transcribing genes. Both the high rate of replication initiation events near highly transcribing genes and the premature depletion of nucleotides create a hotspot for R-T collisions, causing fork stalling that leads to fork collapse and overall genome instability [76,77,78].

## 4. Prevention Mechanisms to Avoid R-Loop Accumulation

Persistent and unhindered R-loops pose a threat to genome stability, therefore, it is fundamental for cells to maintain a homeostasis of R-loop abundance and to prevent their uncontrolled accumulation [9]. This regulation is crucial to maintain the roles of R-loops in their physiological processes and, at the same time, to minimize their pathological impact on genome stability. Alteration of this homeostasis is a major driver of genome and chromosome instability, hallmarks of oncogenesis and neurodegenerative diseases [51,63]. The prevention mechanisms largely act at the level of transcript regulation. Early investigations in budding yeast showed that the perturbation of mRNA biogenesis at any level promotes R-loop formation and DNA damage, thus the natural processing of transcripts acts as a first line of defense against unscheduled R-loop formation [79].

The conserved THO/TREX complex, the first to be characterized in association with transcription-induced R-loops [80], is responsible for nascent pre-mRNA processing with RNA-Binding Proteins (RBPs) to promote the export of the transcript from nucleus to cytoplasm. In yeast and human cells lacking functional THO, mRNA export and transcription elongation are impaired, leading to increased recombination and R-loop accumulation [19,81,82]. This transcription- and R-loop-dependent genome instability highlights the importance of RNA-binding and RNA-processing factors in preventing R-loop accumulation, suggesting that a faulty messenger ribonucleoprotein (mRNP) can bind to the DNA template during transcription, generating detrimental R-loop accumulation [83].

A similar phenotype is also observed for other RNA-processing factors, notably the Serine-Arginine (SR) Splicing Factor (SRSF1) [84]. In higher eukaryotes, splicing is an essential step of mRNA processing and gene expression. SR proteins are pre-mRNA splicing factors that play several roles in RNA metabolism. Depletion in vivo of SRSF1 results in an increased accumulation of DSBs and R-loops. It has been suggested that SRSF1 is recruited to the nascent transcript by RNAPII to prevent formation of mutagenic R-loops [84].

Additional mechanisms to thwart the unscheduled formation of R-loops include the regulation of DNA superhelicity to relieve torsional stress. Torsional stress is known to impede the progression of both DNA and RNA polymerases and can trigger R-loop formation [53,85,86]. Increased negative supercoiling on the trailing fork of the transcription bubble extends the opportunity for potential interactions between newly transcribed RNA and the template strand. DNA topoisomerases relax the negative supercoiling by cleaving the single or the double stranded DNA which has been associated with the prevention of R-loop formation [85,87]. The DNA topoisomerase 3 beta (TOP3B), which has been studied as a component of the Tudor domain-containing protein 3 complex (TDRD3), acts as a methylarginine effector that facilitates gene transcription. TRDR3-TOP3B complex binds to the *c-Myc* promoter to induce *c-Myc* expression. In the absence of TRDR3, the increased levels of R-loops drive *c-Myc/IgH* translocation. TOP3B, by reducing negative supercoiling and preventing R-loop accumulation, protects against DNA damage and reduces the frequency of chromosomal translocations [88]. Moreover, the DNA topoisomerase 2-alpha (TOP2A) and the DNA topoisomerase 2-beta (TOP2B) have been associated with the modulation of DNA topology at the interface between replication and transcription, possibly preventing R-loop accumulation [89,90]. TOP1 seems to be an evolutionary conserved key player in preventing R-loop formation. From bacteria to yeast and mammals, loss of TOP1 leads to enhanced negative supercoiling of DNA behind the polymerases, promoting DNA unwinding and hybridization of the nascent transcript with the DNA template, leading to R-loop accumulation [91,92,93]. A high-resolution strand-specific R-loop mapping in human cells depleted of DNA topoisomerase 1 (TOP1), demonstrated that TOP1 depletion unexpectedly results in both gain and loss of R-loops, defining two different genomic contexts. Surprisingly, the gain of R-loops was found in both highly transcribed as well as heterochromatic regions, whereas the loss of R-loops was associated with open chromatin in moderately transcribing regions, highlighting the fact that chromatin structure and epigenetic context are essential to the regulation of replication stress and are determinants of unscheduled R-loops formation [94].

## 5. Resolving Mechanisms to Remove R-Loops

When regulatory mechanisms fail to prevent R-loop accumulation, the excess R-loops require resolution pathways for their removal. The nuclease activity of Ribonuclease H (RNase H1 and RNase H2), enzymes that are highly conserved from bacteria through metazoans and mammals, in degrading the RNA in RNA:DNA hybrids is crucial to this process [25]. Both enzymes share the essential Hybrid Binding Domain (HBD) through which they bind the RNA:DNA hybrid and degrade the RNA moiety via endonuclease activity. Mammalian RNase H1 has two isoforms: a nuclear isoform and a mitochondrial isoform [95]. The mitochondrial isoform is essential for mitochondrial DNA replication [96] and its depletion has been associated with Chronic Progressive External Ophthalmoplegia (CPEO), a mitochondrial disease [97,98]. RNase H1 also acts on R-loops formed during transcription and plays a role in transcription termination by degrading RNA:DNA hybrids at G-rich pause sites located downstream of the poly(A) site and behind the elongating RNAPII [37]. RNase H2 however, can recognize and cleave misincorporated ribonucleotides in the DNA duplex as wells as remove RNA primers within Okazaki fragments during DNA synthesis [25,99]. The mutations found in RNase H2 have been associated with Aicardi-Goutières Syndrome (AGS), a neuro-inflammatory condition, directly connecting R-loop misregulation with neurodegeneration [36,100]. While RNase H2 can process R-loops created during DNA replication and repair and is possibly cell cycle regulated, as suggested in yeast, RNase H1 can function independently of the cell cycle to remove R-loops and appears to become activated in response to high R-loops loads [101].

Even though the mechanism of RNase H1 recruitment to the site of RNA:DNA hybrids remains unknown, recent studies have explored an intriguing link between the N^6^-methyladenosine (m^6^A) modification on mRNA and the resolution of R-loops via recruitment of RNase H1. m^6^A is a common and reversible post-transcriptional RNA modification, playing a role in various physiological processes, such as gene expression by affecting mRNA splicing, export and degradation, as well as cellular processes, such as immune system and DNA damage responses [102,103]. A recent study using novel m^6^A DNA immunoprecipitation sequencing (DIP-seq) method in parallel to S9.6 DNA–RNA immunoprecipitation sequencing (DRIP-Seq), in human induced Pluripotent Stem Cells (iPSCs), revealed that most RNA:DNA hybrids are m^6^A-modified [104]. These findings are partially corroborated by another recent study, in which only a subset of RNA:DNA hybrids are enriched in m^6^A modification [105]. Furthermore, a transcriptional factor, Tonicity-responsive Enhancer Binding Protein (TonEBP), that regulates cellular osmotic pressure has been suggested to serve as an upstream sensor to bind R-loops and also Methyltransferase-like protein 3 (METTL3), a m^6^A methylation writer, at R-loop site. RNase H1 is then recruited to resolve the R-loops, and its recruitment is dependent on METTL3 catalytic activity. The TonEBP-METTL3-m^6^A methylation is a novel pathway that induces R-loops removal by the recruitment of RNase H1, the absence of which leads to enhanced replication stress and slower cellular proliferation [106].

The R-loops can also be actively processed into DSBs by factors of the Transcription-Coupled Nucleotide Excision Repair (TC-NER) pathway. One such factor, the endonuclease Xeroderma Pigmentosum group G (XPG), was observed to play a role in R-loop processing both in yeast and human, together with other factors of the same pathway [107]. In recent studies, Rad52 is suggested to be recruited at the site of RNA:DNA hybrid formation where it, in turn, recruits XPG to initiate Transcription-Associated Homologous Recombination Repair (TA-HRR), demonstrated by in vivo as well as in vitro methods [108,109]. Rad52 and XPG therefore work in concert to process R-loops after DSB induction. The fact that R-loops are processed by many distinct mechanisms, it is likely that R-loops form at any stage of the cell cycle. However, to preclude their accumulation, cells use other conserved mechanisms that are normally active during a specific cell cycle stage. Possibly, TC-NER factors have a role in recognizing and resolving R-loops associated with paused transcription, mainly in G1 cells.

**Figure 2:**
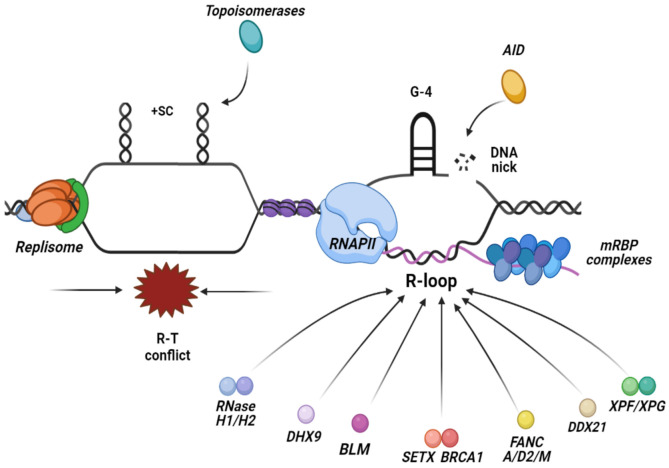
R-loops are formed at the site of collisions between the replisome and RNAPII when they move along the DNA in opposite directions, leading to a head-on conflict between the two machineries. The main preventative mechanisms that inhibit R-loops accumulation are the regulation of torsional stress via the topoisomerases activity that relaxes supercoiled DNA in the vicinity of the forks and the transcript regulation, ensuring the proper packaging and processing of the mRNA. Factors implicated in the resolution of R-loops include nucleases such as, RNase H1/2 enzymes that hydrolyze the RNA moiety of the RNA:DNA hybrid and TC-NER factors, and a plethora of RNA/DNA helicases that unwind the hybrid.

Similarly, there are other factors and pathways that exist during DNA replication in the S phase of the cell cycle, ensuring the resolution of frequent R-T conflicts, mostly through the unwinding activity of RNA/DNA helicases. Among several helicases, there are a few that have relevance in resolving R-loops and discussed below, such as the DEAD-box family helicases DDX21 [110], DHX9 helicase [111], BLM helicase [112], the helicases from the Fanconi Anemia (FA) pathway [113] and the human SETX helicase [56,114] (Figure 2).

The DEAD (Asp-Glu-Ala-Asp)-box family of RNA helicases represents a class of RBPs that are involved in many fundamental aspects of RNA metabolism such as transcription, mRNA transport and RNA decay [115]. There is an emerging body of evidence indicating that several members of the DEAD-box family are multifunctional, playing important roles in both transcription regulation and R-loop homeostasis [110]. The DDX21 helicase is localized in the nucleolus and is required for processing 20S rRNA to 18S. Upon DDX21 knockdown, R-loop formation is enhanced in association with RNAPII stalling and γH2AX foci accumulation. These observations implicate the role of DDX21 in resolving R-loops and guarding against genome instability [110]. Moreover, DDX21 was also demonstrated to reduce estrogen-induced R-loops in breast cancer cells and to moderate replication stress in neural crest and melanoma cells [110,116].

Similarly, DEAD-box helicase DHX9, which plays a critical role during transcription in mediating the interaction between RNAPII and transcription co-activator p300 [117] and tumor suppressor BRCA1 [118], is also involved in unwinding RNA:DNA hybrids and other secondary structures such as G4s [119]. A recent study suggests an association between DHX9 and PARP1 in preventing R-loop-associated DNA damage [111].

Another helicase involved in R-loop processing is the Bloom helicase (BLM). Studies in budding yeast have shown that loss of BLM ortholog Sgs1 makes cells prone to replication-transcription collisions, enhancing R-loop accumulation, which is associated with DNA damage accumulation and copy number changes [112]. Furthermore, in human cells, BLM has been detected in close proximity to RNA:DNA hybrids where it is suggested to play critical role in preventing R-loop-associated genome instability [112]. Moreover, BLM is also found in association with BRCA1 at the site of recombination-based Alternative Lengthening of Telomeres (ALT), where the loss of either of these two factors leads to telomeric dysfunction [120]. A recent study also notes the link between BLM and the FA pathway. It has been suggested that FANCM limits ALT activity in unwinding telomeric R-loops and thus suppressing their accumulation, which can be induced by BLM deregulation [121].

The FA pathway, apart from its role in inter-strand crosslinks (ICLs) DNA repair, has been widely studied for its crucial activity in R-loop resolution and in maintaining concomitant genome stability [113]. In both human cell lines and in primary bone marrow murine cells, loss of either FANCD2 or FANCA leads to R-loop accumulation [122]. Moreover, among the 22 factors identified in Fanconi Anemia, two of them stand out: FANCS, better known as BRCA1, and FANCD1 also known as BRCA2. BRCA1 and BRCA2 are tumor suppressors frequently found mutated in breast cancers [123,124]. BRCA1/2 are the principal factors in the maintenance of genome integrity due to their role in various physiological mechanisms of DNA repair, replication fork stability by mediating stalled fork protection, as well as R-loop resolution [9]. BRCA2 regulates transcription elongation by interacting directly with RNAPII to allow the recruitment of the RNAPII-Associated Factor 1 (PAF-1). PAF-1 subsequently promotes a productive elongation that reduces R-loop formation [125]. Depletion of the FA pathway leads to fork stalling, stabilization of R-loops and consequent genome instability [122,126,127]. Moreover, BRCA2 is also directly involved in the prevention of R-loops accumulation via its association with the TREX-2, a complex that regulates mRNP biogenesis and export in concert with THO complex [128]. On the other hand, BRCA1 has been shown to be involved in the recruitment of SETX RNA/DNA helicase at transcription termination pause sites to resolve R-loops. The BRCA1/SETX complex also plays a key role in restraining the development of R-loop-mediated DNA damage [129].

The role of SETX in transcription termination has been extensively studied in budding yeast and mammalian cells [37,130]. Recently, its role also emerged in replication as it was discovered that budding yeast Sen1, the ortholog of SETX, a bona fide partner of the replisome [131], can suppress R-loop accumulation upon HO R-T conflicts [56]. These findings could be connected as RNA:DNA hybrids accumulation, as well as accumulation of aberrant DNA replication intermediates, can be seen in *sen1* mutants [57]. Furthermore, dysregulation of human SETX has been associated with both neurodegeneration and oncogenesis [114,132]. The role of SETX is seen in two juvenile-onset neurodegenerative disorders: Ataxia with Oculomotor Apraxia type 2 (AOA2) [133] and Amyotrophic Lateral Sclerosis type 4 (ALS4) [134]. AOA2 is linked to the degeneration of the Purkinje cells in the cerebellum which is also commonly associated with ataxia. In this case, *SETX* mutations tend to be recessive frameshift and non-sense mutations, thus associating AOA2 with a loss-of-function of *SETX* [135,136]. In AOA2, the loss of SETX leads to unscheduled persistent R-loop accumulation inducing DNA damage [137]. ALS4 is caused by the degeneration of motoneurons both in the brain and the spinal cord leading to fatal muscle atrophy and, in this disease, the *SETX* mutations are dominant and usually missense, thus associating ALS4 with a gain-of-function of *SETX* [135]. In ALS4 there is a clear disruption of R-loop homeostasis as seen in the missense mutation L389S in ALS4 patients, which is a gain-of-function mutation that results in enhanced activity of SETX helicase in the removal of R-loops. Such a mutation alters R-loop homeostasis due to the loss of R-loop-mediated gene silencing, which results in the alteration of the expression of several key genes, including the BMP and Activin Membrane-Bound Inhibitor (BAMBI) of Transforming Growth Factor beta (TGFβ) signaling pathway, commonly altered in motor neuron diseases [49,138]. SETX role in oncogenesis is linked to the physical interaction with BRCA1 and its role in R-loop resolution and DNA repair. BRCA1 mediates the recruitment of SETX at R-loops to form a transcriptional terminator pause site allowing the removal of R-loops [129]. Mutations in SETX and BRCA1 are linked to DNA damage and gross genome rearrangements at the points of gene termination, a common feature of the BRCA tumors. The importance of BRCA-FA-SETX in preventing R-loop-mediated DNA damage and genome instability provides strong evidence of the oncogenicity of the R-loop structures [122,129].

## 6. The Enigmatic Role of RNA:DNA Hybrids in DNA Repair

For decades, unscheduled formation of R-loop/RNA:DNA hybrids has been considered as cause of DNA damage and genome instability. However, recent studies have suggested a rather fascinating role for transient RNA:DNA hybrids formation at sites of DNA damage in facilitating DNA repair. A recent study reports an interaction between BRCA2 and DDX5, a known DEAD-box helicase that also regulates resolution of RNA:DNA hybrids [139], particularly at DSBs sites. It is shown that BRCA2 stimulates the RNA:DNA helicase activity of DDX5 favoring its association with RNA:DNA hybrids in the vicinity of DSBs and finally promoting Homologous Recombination (HR) repair pathway [140]. Such role for BRCA2 has also been shown in another study that shows how BRCA2 regulates RNA:DNA hybrids levels at the DSBs site by mediating RNase H2 recruitment during S/G2 cell-cycle phase [141].

Moreover, it has been suggested that microRNA (miRNA) biogenesis enzymes, DROSHA and DICER, control the recruitment of repair factors from multiple pathways to sites of damage. DROSHA is suggested to be required for RNA processing within minutes of break induction, thus playing a central role in early stages of DNA repair. In the absence of DROSHA, a significant reduction of DNA repair by both HR and NHEJ was reported [142]. The role of RNA:DNA hybrids in DNA repair was also observed in fission yeast, where deletion of RNase H1 and RNase H2 resulted in the accumulation of RNAPII and RNA:DNA hybrids at the site of DSBs, leading to the inhibition of HR-mediated DSB repair [143]. Similarly, a recent study in budding yeast has associated the Sen1 ortholog with DSBs repair, as previously proposed in human [144]. Cells lacking a functional Sen1 present elevated levels of persistent RNA:DNA hybrids in the proximity of DSBs. At these DSB sites, RNA:DNA hybrids in concert with DNA nucleases Mre11 and Dna2, initiate DSB-resection through a non-canonical mechanism. This mechanism acts as a backup to prime HR repair when the canonical pathway, in which the short-range resection initiation by Mre11-Rad50-Nbs1 (MRN) complex is followed by a nuclease-dependent long-range resection, is hindered. However, persistence of RNA:DNA hybrids at the DSB site might also interfere with the resection process, leading to increased activation of mutagenic repair pathways such as NHEJ and Micro-homology-Mediated End Joining (MMEJ) [35].

Recent reports highlight the potential role of RNA:DNA hybrids, and in particular of the RNA moiety, to regulate the multiple steps of DSB repair, substituting the dsDNA natural substrate. R-loops have been shown to trigger non-canonical and extensive resections [35] and to regulate the RAD51 nucleofilament assembly also through non-canonical mechanisms that rely on Cockayne Syndrome group B (CSB) and RAD52 instead of BRCA1 and BRCA2 [109,145]. R-loops seems to recruit repair proteins underscoring a beneficial role in repair through non-canonical mechanisms which need to be timely and strictly regulated to be ultimately compatible with the unavoidable removal of RNA:DNA hybrids to allow maintenance of genome integrity [146,147].

Moreover, the role of R-loops has also been associated with the checkpoint activation. These mechanisms are detailed in a recent review article [62]. Briefly, the CD conflicts were associated with Ataxia Telangectasia Mutated (ATM) pathway activation while HO conflicts were associated with Ataxia Telangiectasia and Rad3-related protein (ATR) pathway activation, although the nature of this distinction is still unknown [58]. However, a recent study has further shown that loss of ATR, but not ATM, leads to significant increase in R-loop levels that are the ultimate source of replication stress and thus the ATR pathway being primarily required for their resolution [148]. However, this study also implicates that the slight increase in R-loop levels observed upon loss of ATM is possibly a consequence of unrepaired DSBs that accumulate genome wide in absence of ATM rather than a cause of DSBs formation.

## 7. Role of Epigenetic Marks in R-Loop Homeostasis

**Figure 3:**
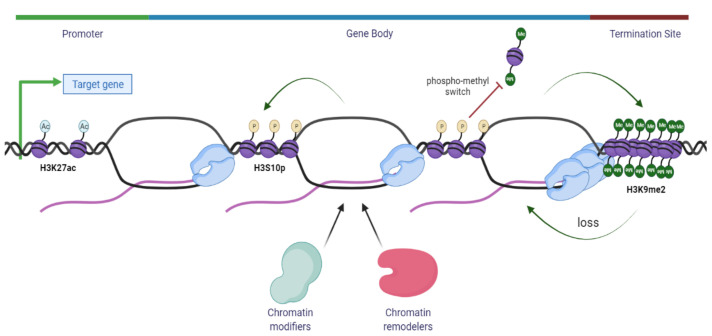
Summary of chromatin modifications associated with the R-loops. R-loops accumulate at the promoter region of genes and are usually associated with an open chromatin structure characterized by H3 acetylation marks, such as H3K27ac and other permissive epigenetic modifications, allowing gene expression and transcription initiation. At terminators, R-loops are formed to induce transcription termination at sites with paused RNAPII, and these R-loops induce the activity of EHMT2/G9a methyltransferase to compact the chromatin by depositing the heterochromatin H3K9me2 mark. Moreover, R-loops are also associated with a closed chromatin architecture that induces repressive marks, such as H3S10p, that might act as a platform for chromatin modifiers and remodelers to alter the chromatin landscape. The antagonism between H3S10p and H3K9me2 prevents the spread of heterochromatin regions across the genome.

An intriguing idea that R-loops may act as an epigenetic layer has also emerged from the fact that R-loops show co-occurrence with specific chromatin epigenetic marks and post-translational modifications (PTMs), in mapped R-loops loci [149,150]. As previously discussed, R-loops are frequently found at unmethylated CpG island promoters [11] and at the 3′-end of several genes [22] where they play a key role in transcription termination [37]. Using an innovative R-loop genome-wide mapping method, DNA:RNA Immuno-Precipitation followed by cDNA conversion coupled to high-throughput sequencing (DRIPc-Seq), R-loops have been shown to associate with specific epigenomic marks at promoters and terminators [23]. At the promoters, R-loops are enriched in histone marks such as methylation of H3, as seen with mono- and tri- methylation of lysine 4 and 36 residues of histone 3 (H3K4me1/3, H3K36me3) and open chromatin marks of histone 3 acetylation, such as the acetylation of lysine 27 residue (H3K27ac), while at terminators, R-loops are associated with H3K4me1, suggesting that H3K4me1 is a common mark of R-loop regions [11,22,23,39]. Even though all these epigenetic marks are associated with increased chromosome accessibility and thus active gene expression, it is intriguing how chromatin modifiers may sense the chromatin status associated with distinct R-loops that further play a role in epigenetic regulation on gene expression [39] (Figure 3).

Previously, R-loops have been linked to chromatin condensation through the epigenetic modification of phosphorylation at serine 10 residue of histone 3 (H3S10p) [151]. An unexpected link is proposed between R-loops, histone modification and chromatin condensation in which R-loops might trigger the formation of highly compact chromatin. This is either by promoting phosphorylation of Serine 10 residue of histone H3 or by inhibiting its dephosphorylation. It has been suggested that such condensation could spread throughout the genome, leading to enhanced R-T collisions and gene silencing associated with genome instability [151]. Therefore, pathological R-loop formation seems to be associated with H3S10p and chromatin condensation, an association that is in opposition to the link between R-loops and active, hyper-accessible chromatin under physiological conditions [39]. Further, histone modification H3S10p is unique because of its involvement in two opposing processes: transcription activation and chromatin condensation during the cell cycle, as this epigenetic mark increases on mitotic chromosomes instead of being erased upon entry to mitosis [152]. Therefore, a possibility might be that H3S10p, rather than having a direct action on chromatin status, merely acts as a platform to recruit chromatin modifiers and remodelers. There are several nuclear kinases involved in the phosphorylation of H3S10p and this epigenetic mark has been demonstrated to inhibit the histone lysine methyltransferase (KMT) activity of Su(var)3-9 family members such as SUV39H1 and EHMT2/G9a [153]. EHMT2/G9a and EHMT1/G9a-like protein (GLP), amongst other histone methyltransferases, deposit the H3K9me2 mark on euchromatic regions in mammals, while SUV39H1 is associated with the maintenance of constitutive heterochromatin regions [154].

In particular, the ankyrin repeats of G9a and GLP are inhibited by H3S10p, creating an antagonism between H3S10p and H3K9me2 that has been called the “phospho-methyl switch” [155]. The interplay between H3S10p and H3K9me2 has been documented in *Drosophila melanogaster*, where in the presence of the JIL-1 hypomorph, the main H3S10p kinase, there is a wide-spread loss of euchromatic interbands with propagation of H3K9me2 heterochromatin bands, reinforcing the concept that H3S10p delimits the boundaries of euchromatin in flies by antagonizing heterochromatin propagation [156,157]. These findings have been recently demonstrated also in Mouse Embryonic Fibroblasts (MEFs) and Mouse embryonic stem cells (ESCs) where this antagonism highlights the role of H3S10p in inhibiting the deposition of heterochromatin H3K9me2 mark in mammals [153]. Moreover, transient formation of H3K9me2 has also been shown to accumulate at the terminator regions of genes at RNAPII pausing sites. At the terminator regions, R-loops induce antisense transcription which leads to the formation of dsRNA. This leads to the recruitment of RNAi pathway factors along with lysine methyltransferase EHMT2/G9a, that mediate the formation of repressive epigenetic mark H3K9me2, further reinforcing the pause of RNAPII [150]. This illustrates a direct link between R-loop activity and chromatin architecture modification in which additional chromatin remodelers and modifiers are implicated. Among the many remodelers and modifiers that are thought to have a role in R-loop homeostasis, we highlight a few relevant ones in Table 1.

## 8. The Role of the Chro-Mates Part I: Chromatin Modifiers

### 8.1. EHMT2/G9a

R-loops or RNA:DNA hybrids are linked to condensed chromatin as indicated by repressive chromatin markers such as H3K9me2 and H3K9me3 shown in *Caenorhabditis elegans* [177] and in human [150]. While de-condensation of chromatin has been linked to accumulation of R-loops [178], it is not clear if R-loops are the cause or the consequence of the chromatin changes. Indeed earlier studies have shown that RNA:DNA hybrids can lead to formation of compact chromatin in yeast and human [150,179]. It has been suggested that RNA:DNA hybrid-induced antisense transcription, subsequently lead to recruitment of G9a and form the H3K9me2 repressive mark. Furthermore, RNase H1 over-expression could suppress RNA:DNA hybrids as well as H3K9me2 at 5′-end pause regions, while G9a inhibition might only suppress the H3K9me2 signal, but not R-loop levels [150]. This suggests that RNA:DNA hybrids may be the cause of the change in the epigenetic mark. Moreover, recent studies also reported that G9a inhibition suppresses H3K9me2 and leads to the accumulation of R-loops in ribosomal DNA (rDNA) sites [158]. These findings suggest multiple roles of G9a in the regulation of R-loop formation by governing chromatin compaction.

### 8.2. SIRT6

In budding yeast, the histone acetyltransferase (HAT) complex Rtt109 promotes acetylation of H3K56 (H3K56ac) during the S-phase of cell cycle, causing a weaker interaction between histone H3 and DNA. It has been suggested that this modification promotes a more accessible chromatin conformation that favor fork progression [180,181]. Such acetylation is antagonized by mammalian tumor suppressor SIRT6 deacetylase. SIRT6 is part of the Sirtuin family of conserved NAD^+^-dependent lysine deacetylases [182,183], and has been shown in mice models to serve also as a tumor suppressor [184]. Hst3 and Hst4, homologues of SIRT6 in budding yeast, regulate histone H3 deacetylation and repress nascent transcription as well as co-transcriptional R-loop formation.

In the absence of Hst3 and Hst4 a global increase in nascent transcripts was observed, possibly due to increased histone H3K56ac levels that creates a favorable environment for transcription initiation. However, in these double mutants an increased number of RNA molecules at the 5′-end compared to 3′-end and gene body were found. This suggested that although transcription initiation is promoted, RNA polymerases are not entering a robust elongation phase [159]. This is consistent with previous studies demonstrating that the deletion of Hst3 and Hst4 is associated with numerous genome instability phenotypes, and that these phenotypes are suppressed by the inactivation of the Asf1 subunit of Rtt109 HAT complex [160,185]. Therefore, a prolonged hyper-acetylation of H3K56 in cells depleted of histone deacetylase, SIRT6/Hst3-Hst4 causes the replication machinery to interact with a more open and transcriptionally accessible chromatin ahead of the forks, creating a barrier that leads to enhanced formation of transcription-associated R-loops [159,160,185].

### 8.3. SIRT7

As previously discussed, members of the DEAD-box family of RNA/DNA helicases are involved in R-loop resolution, RNA metabolism and in the maintenance of genome stability. Members of this family are DDX5, DDX19, DDX23 and DDX21 [186]; the latter has been described above. The enzymatic activity of DDX21 is, however, regulated by acetylation-deacetylation of this protein. cAMP-response element Binding Protein (CBP)-mediated acetylation of DDX21 inhibits its enzymatic function, while DDX21 deacetylation mediated by NAD^+^-dependent deacetylase SIRT7 improves R-loop unwinding activity [110]. Therefore, a possible functional link between DDX21 and SIRT7 activity seems to exist, and this is further corroborated by findings suggesting that depletion of either DDX21 or SIRT7 results in enhanced R-loop accumulation, DNA damage and genome instability [110]. The role of SIRT7 in establishing an interaction with chromatin remodeling complexes of the SNF2 family has been noted [187], therefore its role in chromatin remodeling and modification has been explored. This additional role in conjunction with DDX21 further underlines the importance of chromatin structure in the resolution of R-loops. Moreover, SIRT7 has also been studied in mice, where *Sirt7*^−/−^ knockout induces partial embryonic lethality and premature aging, reaffirming its importance to genome stability [161]. The results of other studies have indicated that SIRT7 by itself has a role in genome stability and R-loop resolution, however, not necessarily due to its deacetylase activity. Indeed, SIRT7 also catalyzes H3K122 histone desuccinylation at DSBs sites [162]. H3K122 is located on the lateral surface of the histone octamer, and PTMs in this region facilitate nucleosome mobilization and eviction, modulating chromatin accessibility. Desuccinylation has been linked to chromatin condensation, which has been previously associated with R-loop accumulation [151,162].

### 8.4. Sin3A

As described above, THO/TREX complex is an essential factor that mediates proper nascent transcript packaging and export into the cytoplasm [80,82,83,188]. However, it seems that the ability of the THO complex to suppress R-loop accumulation is not solely due to its role in mRNP assembly. Through interaction studies of THO complex, it was observed that human THOC1, a subunit of the THO complex, physically interacts with the histone deacetylase Sin3A [163]. It was further demonstrated that depletion of Sin3A complex leads to R-loop stabilization and DSB accumulation, consistent with previous findings in *sin3∆* mutants in budding yeast [189], as well as the phenotypes observed upon THO depletion [81]. The Sin3A and THOC1 knock-down cells show faster fork progression rates, however, an increased frequency of fork stalling was also detected due to fork asymmetry. The stalling of replication forks is caused by the impediment of R-loops, since fork asymmetry was suppressed upon RNase H1 over-expression [163]. These observations indicate that hyper-acetylation leads to an open chromatin that facilitates fork progression. However, such hyper-accessible chromatin is a hotspot for R-loop accumulation, this also poses as an obstacle to replication forks progression [23]. This interaction between THO and Sin3A might suggest a novel mechanism of R-loop resolution in which RNA biogenesis for transcript regulation and cross-talk with chromatin modifiers resulting in chromatin compaction, would avoid excessive R-loop formation [163]. It is imperative to highlight not only that hyper-accessible chromatin is associated with RNA:DNA hybrid or R-loop accumulation, but that it is also associated with compact chromatin, as previously discussed with H3S10p and H3K9me2 epigenetic marks [149,150,151]. This discrepancy could imply that the heterochromatin marks play a role in stabilizing RNA:DNA hybrids that are formed in an open chromatin environment and may contribute to genome instability.

### 8.5. RNF168

**Figure 4:**
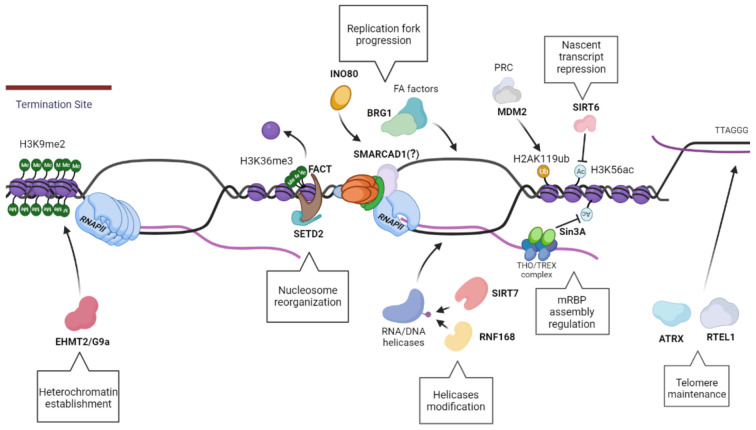
Schematics showing multiple distinct pathways of R-loop resolution mediated by the Chro-Mates, the chromatin modifying and remodeling factors, described in Section 8 and Section 9. EHMT2/G9a regulates the preservation of the heterochromatin, especially at the termination sites, in order to permit a successful transcription termination. In addition, the nucleosome reorganization upon RNAPII passage is a vital pathway in R-loops resolution, driven by FACT in concert with SETD2. The modification of RNA/DNA helicases has a role in their recruitment and processivity, such as RNF168 ubiquitinates DHX9, while SIRT7 deacetylates DDX21. The homeostasis of the nascent transcript is also essential to regulate R-loop levels, as seen with Sin3A and SIRT6, histone deacetylases that have a role in creating an optimal chromatin status for a proper transcript export and maturation, along with fork progression. Moreover, the integrity of telomeres is preserved via ATRX and RTEL1, which promotes telomere stability and R-loops resolution. Finally, one of the most critical aspects of R-loop resolution is maintenance of fork stability and replication fork progression. This may require factors involved in resolution of R-loops: as seen with MDM2, that promotes fork progression and gene expression; INO80 possibly resolve R-loops by inducing chromatin relaxation; BRG1 works in concert with FA factors to create an open chromatin structure for the recruitment of R-loops resolvases; and Fft3, which can resolve R-loops in fission yeast with its ortholog SMARCAD1 traveling with the replication forks.

Ring Finger protein 168 (RNF168) is a ubiquitin E3 ligase that plays a critical role in DNA damage repair pathway. RNF168 is well known to mediate K63-mediated ubiquitination of p53 Binding Protein 1 (53BP1) and of H2A histones at K13-15, both required marks for recruitment and retention of 53BP1 at DNA damage sites [190,191]. However, a recent study has demonstrated that loss of RNF168 results in R-loop accumulation in *BRCA1/2*-mutant cancer cells, leading to DSBs accumulation, senescence and cell death [164]. In this study, using interactome assays, RNF168 was identified as interacting partner with DHX9, an RNA/DNA helicase that is involved in the resolution and removal of R-loops, as mentioned above [111,164]. RNF168 directly ubiquitinates DHX9 at K697 and K708 aiding its recruitment at R-loop-forming loci. The loss of RNF168 results in an impairment of DHX9 activity, thus abrogating its role in R-loop resolution. Moreover, using mouse models it was shown that Rnf168 depletion protects against *Brca1*-mutated mammary tumorigenesis, and this data is further corroborated by the identification of a human genetic variant that reduces breast cancer risk in *BRCA1*-mutation carriers when they are associated with reduced RNF168 expression levels [164]. Together, these results bring to light the existence of a factor that indirectly, by promoting DHX9 recruitment, resolves R-loops accumulation (Figure 4).

There are increasing number of studies focused on the identification of co-factors regulating the helicases/nucleases [192]. However, very little is known about their regulation by PTMs and epigenetic modifications that facilitates the direct processing of R-loops. This field of research requires further studies focusing on identifying mechanisms, acting uniquely at distinct stages of cell cycle.

## 9. The Role of the Chro-Mates Part II: Chromatin Remodelers

### 9.1. MDM2

MDM2 is historically known as the negative regulator of p53 [193]. However, as a chromatin remodeler, MDM2 enhances H3K9me3 levels [194] and modulates Polycomb Repressor Complex (PRC)-driven histone modifications H2AK119ub1 and H3K27me3, with both RING1B (PRC1) and EZH2 (PRC2) [195]. H2AK119ub1 is frequently associated with the regulation of gene expression. Furthermore, loss of MDM2 or its E3 ubiquitin-ligase domain results in reduced H2A ubiquitination levels and is associated with slower replication fork progression. Over-expression of the deubiquitinating enzyme BRCA1-associated protein1 (BAP1) is shown to promote fork progression in wildtype, but not in the MDM2 depleted cells, suggesting that timely ubiquitination, as well as deubiquitination, are critical for replication fork progression [165]. MDM2 depleted cells also shows enhanced R-loops accumulation and genome instability, an indication of MDM2 many roles in regulating condensed chromatin. Even though chromatin modification status in relation to R-loops isn’t clear in MDM2 depleted cells, over-expression of RNAse H1 rescues replication defects, suggesting the possibility of a link between ubiquitination of H2AK119 and R-loop formation [165].

### 9.2. ATRX

ATRX is a member of the SWItch/Sucrose Non-Fermentable (SWI/SNF) family of chromatin remodeling factors that localize to rDNA repeats, telomeric repeats, pericentromeric repeats and minisatellites [166]. Loss of ATRX affects cellular processes such as methylation [196], gene expression [197] replication and the maintenance of genome stability [166]. In many cancer cells, telomere shortening is prevented either by an abnormal telomerase activity or by the induction of the ALT pathway, briefly described above (for reviews see [198,199,200,201]). The telomeric-repeat-containing RNA (TERRA) plays a critical role in regulating the telomerase [202,203]. Since TERRA acts as a competitive inhibitor of telomeric DNA, at this site there is a notable accumulation of R-loops which induces DNA damage and recombination [204]. Moreover, TERRA has also been seen having an important function in the epigenetic modification of telomeres [205]. TERRA promotes gene expression at telomeric DNA by strongly binding the ATP-dependent helicase and chromatin remodeler ATRX, thereby preventing ATRX-dependent gene suppression. TERRA and ATRX are functionally antagonistic at telomeric sites where TERRA directly binds ATRX, displacing it, thus making the relationship between TERRA and ATRX essential for telomere maintenance and protection [167,206,207]. It is known that the ALT pathway is involved in the suppression of ATRX [207], and a recent study has revealed that the loss of ATRX causes higher accumulation of R-loops in telomerase-positive cells. Moreover, re-introduction of ATRX reduced R-loop levels in ALT-positive cells [166]. These observations suggest an intriguing role for telomere regulation in R-loop resolution and homeostasis. The retention of R-loops at telomeric regions although remains unclear, however, a recent study reports that Regulator of Telomere Length 1 (RTEL1) helicase regulates the levels of TERRA RNA and thus, plays a key role in the maintenance of TERRA-containing telomeric R-loop and telomere stability [208]. It is shown that loss of RTEL1 leads to an accumulation of RNA:DNA hybrids and G4s structures forming at the displaced ssDNA and a consequent exacerbation of R-T conflicts, generating replication stress at common fragile sites and also causing telomere instability [209,210].

### 9.3. FACT/SETD2

Nucleosomes pose a physical impairment to transcription as first discovered via in vitro study [211]. It was shown that nucleosome-coated DNA sterically denies access to auxiliary factors and thus affects in vitro transcription on a chromatinized DNA template [212,213]. DNA replication and transcription are facilitated by several histone chaperones, and one of the critical chaperones is the Facilitates chromatin transcription (FACT) complex that aids in both these processes. FACT is a heterodimer complex composed of Spt16 and SSRP1 in most eukaryotes or Pob3 in budding yeast [214,215]. FACT promotes RNAPII transcription elongation by disrupting nucleosomes, weakening the link between DNA and H2A-H2B dimers and later reassembling them. FACT also contribute to replication-coupled nucleosome assembly and might travel with the replication fork due to its interaction with some core replisome machinery factors [216,217,218]. FACT cooperates with chromatin assembly factor-1 (CAF-1), a histone chaperone that promotes deposition of H3-H4 tetramers into DNA by interacting with proliferating cell nuclear antigen (PCNA) and anti-silencing function 1 protein (ASF1), another histone chaperone to reorganize chromatin on nascent DNA [181]. Furthermore, FACT is also known to maintain heterochromatin integrity via its interaction with heterochromatin protein 1 (HP1) in mammals or Swi6 in fission yeast, at pericentromeric regions [219,220].

A study conducted in yeast FACT mutants and FACT-depleted human cells shows that the complex plays a key role in allowing fork progression upon R-loop-dependent R-T conflicts [168]. Moreover, it has also been shown that in these mutants there is an accumulation of Rad52 foci and R-loops, leading to DNA damage and genome instability [168]. As it is established, R-loops promote chromatin compaction and heterochromatinization through the deposition of epigenetic marks such as H3S10p and H3K9me2, shown in yeast, worm, flies and human [150,151,157], it is likely that FACT might promote a chromatin conformation that suppresses R-loop accumulation thereby allowing normal fork progression and transcription elongation. It is also noted that FACT works in concert with SETD2, a histone methyltransferase responsible for the H3K36me3 epigenetic mark in active genes. Depletion of yeast Set2 and downregulation of human SETD2 reduces FACT recruitment at active genes, thus affecting nucleosome organization [169]. In addition, SETD2 promotes a reduction in histone H2B levels, enhancing nucleosome reorganization along with FACT. These observations suggest the possibility of SETD2-dependent H3K36me3 at active genes as a docking site for FACT to induce proper nucleosome reformation to deposit H2A-H2B dimers after RNAPII passage [169]. This model is further supported by the knowledge that FACT activity is influenced by several PTMs such as ubiquitination [221]. Thus, not only FACT, but also SETD2 are required to create an optimal chromatin structure at active transcription sites in order to prevent the accumulation of R-loops and transcription-replication conflicts, thus reducing the R-loop level via a feedback loop.

### 9.4. INO80

Even though we have discussed factors involved in chromatin compaction and condensation as a pathway to resolve R-loops, factors that relax chromatin may also resolve R-loops. A recent study indeed describes INO80 as a factor that suppresses R-loops by decompacting and relaxing the chromatin around the R-loops structures [170]. In this study, the role of INO80 in facilitating DNA replication, averting R-T collisions and inhibiting co-transcriptional R-loops is elucidated. The INO80 complex is a well-characterized chromatin remodeler [222] that has been demonstrated in budding yeast to suppress R-loops by promoting the extraction of ubiquitinated RNAPII, thus removing the major obstacle to fork progression [171,223]. Moreover, human INO80 has been described as a remodeler that opens the chromatin structure [224] and many studies demonstrate compact chromatin in the R-loop surrounding region [149,150,151]. A recent study employing LacO array designed to accumulate RNA:DNA hybrids and concomitant replication stress, showed that artificial tethering of INO80 resolves R-loops. Furthermore, this study highlights the importance of the INO80 complex in promoting the resolution of R-loops to prevent replication-associated DNA damage in cancer cells to promote their proliferation [170].

### 9.5. BRG1

SWI/SNF chromatin remodelers have an important role in remodeling the chromatin by mobilizing the nucleosomes, displacing and re-inserting the histone octamer [225] and these factors have been shown to be mutated in approximately 20% of human cancers [226]. The human analog of the SWI/SNF family is the BRG1-associated factor (BAF) and its various subtypes such as the canonical BAF (cBAF), non-canonical BAF (ncBAF); these factors share in common the main SWI/SNF catalytic subunit, BRG1. These complexes are well known for their nucleosome remodeling activity that includes sliding of nucleosomes, eviction and insertion of histone octamers to maintain high-order chromatin structure [225,227].

Strikingly, BRG1 is one of the most frequently mutated chromatin remodeling factors found in various cancers. A recent study demonstrates that BRG1 plays a critical role in the regulation of R-loops and R-loops-dependent R-T conflicts [172]. BRG1 is shown to co-localize with R-loops that block replication forks and recruit FANCD2, while BRG1 and FANCD2 loss is epistatic to R-loop accumulation [172]. Their findings show transient depletion of BRG1 in HeLa cells resulted in substantial increase in R-loops accumulation which is further accompanied with slower fork progression and R-loop-dependent DNA breaks. This study strongly suggests a role for other SWI/SNF remodelers, such as PBRM1 and ARID1A, in regulating chromatin structure at R-loop-derived R-T conflict sites, allowing a more accessible and open chromatin structure that might permit the binding of known factors involved in R-loop resolution such as SETX, RNase H1, BRCA2 and FA.

### 9.6. Fft3/SMARCAD1

SMARCAD1, a DEAD/H box helicase domain protein, belongs to the highly conserved ATP–dependent SWI/SNF family of chromatin remodelers. The budding yeast ortholog, FUN30 has been shown to directly bind ssDNA, dsDNA and nucleosomal DNA, in vitro [228,229]. Fun30 has been shown to slide and reposition nucleosomes as well as to exchange H2A-H2B histone dimers [229,230]. The nucleosome remodeling activity of SMARCAD1/Fun30 has been suggested to play role in mediating resection to promote HR repair [231,232]. Moreover, the nucleosome remodeling activity of SMARCAD1/Fun30/Fft3 has also been suggested to maintain heterochromatin [173,174,175,230] by promoting H3/H4 deacetylation in order to stably maintain H3K9me3 methylated nucleosomes [174,233,234,235]. Interestingly, in fission yeast, Fft3 is shown to suppress histone turnover at heterochromatin regions to ensure the correct propagation of parental nucleosomes. Fft3 was further shown to suppress histone turn-over at difficult-to-replicate regions in order to preclude RNA:DNA hybrid formation that can lead to impaired fork progression [176]. It was suggested that in absence of Fft3, the enhanced turn-over leads to an open chromatin structure that facilitates R-loop formation at specific loci of the genome, especially those that are highly transcribing and/ or contain intragenic repetitive sequences [176].

More recently SMARCAD1 is shown to mediate a novel pathway of active replication fork stability by maintaining PCNA homeostasis at forks, distinct from BRCA-mediated stalled fork protection pathway [176]. Given that many of the factors discussed, apart from having a role in DNA breaks repair also have a role in replication fork stability and remodeling pathways [176,236,237,238,239,240,241], it is possible that chromatin remodelers such as SMARCAD1 could have a role in the resolution of R-loops, as previously suggested in the fission yeast ortholog Fft3 [175]. Moreover, since SMARCAD1 travels with the replisome, its action could regulate R-loop resolution ahead of the fork, an interesting possibility that is yet to be tested.

## 10. Concluding Remarks

Since their discovery, R-loop structures have garnered interest considering the conserved duality of their role in both replication and transcription contexts. In particular, upon conflict between replication and transcription, an inevitable event further accentuated in difficult-to-replicate regions, R-loops pose as one of the major drivers of genome instability [43,49,51,175]. Replication and transcription require the continuous unwinding and rebuilding of nucleosomes and, in this context, chromatin remodelers and modifiers play a central role. Most of the studies to date have focused on the characterization of prevention and removal pathways of RNA:DNA hybrids, while only in recent years has the role of the chromatin landscape been explored. As described above, there are many factors among chromatin remodelers or chromatin modifiers that have a role in R-loop biology. An interesting and contrasting outcome that emerges from this review is that both chromatin accessibility and chromatin condensation are linked to the presence of RNA:DNA hybrids [23,39,150,151]. Given the present state of knowledge, it appears safe to propose a model in which genome stability is ruled by the maintenance of both R-loop and chromatin accessibility homeostasis, depending upon the distinct territory of a genomic region [23,38,94]. An intriguing model would envision the open chromatin as being a hotspot for the formation of R-loops and subsequently, chromatin compaction playing a role in the accumulation and stabilization of these structures, highlighting the close relationship between RNA:DNA hybrids and chromatin landscape.

Furthermore, the importance of a fine regulation of R-loop levels is essential to genome integrity, as not only the scheduled but also the unscheduled, yet transient, RNA:DNA hybrids potentially play a role in physiological processes protecting genome integrity. Examples of the latter include the transient RNA:DNA hybrids at the site of DSBs, which are responsible for early stages of DNA repair, efficiently recruiting DNA repair factors. The existence of these structures suggests the interesting possibility that even the unscheduled RNA:DNA hybrids formation must be preserved, as long as they are not persistent and can be timely removed [35,49,142,143,242].

An intriguing unknown mechanism by which various chromatin remodeling and histone modifying factors, associated with distinct state of replication forks and involved in R-loop metabolism, distinguish between the persistent/detrimental RNA:DNA hybrids existing ahead of the fork as an impediment, from the ones associated with the replisome in the form of Okazaki fragments. Furthermore, there might be distinct resolving activities associated with unperturbed and stressed forks, such that the factors associated with unperturbed forks promote fork progression by remodeling the chromatin ahead of the fork, preventing conflicts from occurring, while the resolution of R-loops ahead of stalled fork may involve a distinct mechanism/factor involved in the removal of the conflict and thereby, promote the restart of the fork.

It is therefore crucial to determine if there are distinct chromatin features associated with the resolution of harmful, persistent R-loops and transiently-formed, beneficial R-loops. Furthermore, how and which chromatin remodeling/modifying factors, in concert with distinct resolvases, differentiate between these two kinds, from their recognition to their processing, to the maintenance of fine R-loop homeostasis, is yet to be fully elucidated. A recognition of two such distinct regulatory systems with the identification of their components would open up new avenues of research in which chromatin remodeling and modifying activities as a whole will be studied with a new perspective. Targeting molecular pathways and factors that promote the activity of beneficial R-loops while inhibiting the activity of harmful R-loops offers a promising new avenue for therapeutic intervention in a wide array of R-loop-associated diseases.

## Figures and Tables

**Table 1:** Representative Chro-Mates implicated in the processing of R-loops.

**Chro-Mates**	**Homologues**	**Activity**	**Role in** **R-Loop Processing**	**Ref.**
EHMT2/G9a	DmeI (*Drosophila melanogaster*)	Modifier	Methylation of H3K9me2 at heterochromatin regions	[150,153,158]
SIRT6	Sirt6 (*Mus musculus*) Hst3/4 (*Saccharomyces cerevisiae*)	Modifier	Deacetylation of H3K56ac	[159,160]
SIRT7	Sirt7 (*Mus musculus*)	Modifier	Deacetylation of DDX21 Histone desuccinylation	[110,161,162]
Sin3A	Sin3 (*Saccharomyces cerevisiae*)	Modifier	Histone deacetylation through interaction with THO complex	[163]
RNF168	Rnf168 (*Mus musculus*)	Modifier	Ubiquitination of DHX9	[164]
MDM2	Mdm2 (*Mus musculus*)	Remodeler	Ubiquitination of H2AK119 and genome expression	[165]
ATRX	Atrx (*Mus musculus*)	Remodeler	Antagonization of TERRA RNA at telomeric R-loops	[166,167]
FACT/SETD2	yFACT/Set2 (*Saccharomyces cerevisiae*)	Remodeler	Nucleosome reassembly after RNAPII passage	[168,169]
INO80	Ino80 (*Saccharomyces cerevisiae*)	Remodeler	Chromatin relaxation	[170,171]
BRG1	Swi (*Saccharomyces cerevisiae*)	Remodeler	Regulation of chromatin accessibility	[172]
Fft3/SMARCAD1	Fun30 (*Saccharomyces cerevisiae*) Fft3 (*Schizosaccharomyces pombe*)	Remodeler	Nucleosome turn-over Active fork protection	[173,174,175,176]

## Abbreviations

53BP1	p53 Binding Protein 1
AGS	Aicardi-Goutières Syndrome
AID	Activation-Induced Deaminase
ALS4	Amyotrophic Lateral Sclerosis type 4
ALT	Alternative Lengthening of Telomeres
AOA2	Ataxia with Oculomotor Apraxia type 2
ASF1	Anti-silencing function 1 protein
ATM	Ataxia Telangectasia Mutated
ATR	Ataxia Telangectasia and Rad3-related protein
BAF	BRG1-associated factor
BAMBI	BMP and Activin Membrane-Bound Inhibitor
BAP1	BRCA1-associated protein 1
BER	Base Excision Repair
CAF-1	Chromatin assembly factor-1
cBAF	Canonical BAF
CBP	cAMP-response element binding protein
CD	Co-directional
CFSs	Common Fragile Sites
CPEO	Chronic Progressive External Ophthalmoplegia
CSB	Cockayne syndrome group B
CSR	Class-Switch Recombination
DIP-seq	DNA immunoprecipitation sequencing
DRIP-Seq	DNA–RNA Immuno-Precipitation sequencing
DRIPc-Seq	DNA-RNA Immuno-Precipitation followed by cDNA conversion coupled to high-throughput sequencing
DSB	Double-strand break
ERFSs	Early Replicating Fragile Sites
ESCs	Mouse embryonic stem cells
FA	Fanconi Anemia
FACT	Facilitates chromatin transcription
G4	G-quadruplex
GLP	G9a-like protein
HAT	Histone acetyltransferase
HBD	Hybrid binding domain
HO	Head-on
HP1	Heterochromatin protein 1
HR	Homologous Recombination
ICLs	Inter-strand crosslinks
Ig	Immunoglobulin
iPSCs	induced pluripotent stem cells
m6A	N6-methyladenosine
MEFs	Mouse Embryonic Fibroblasts
METTL3	Methyltransferase-like protein 3
miRNA	microRNA
MMEJ	Micro-homology-Mediated End Joining
MMR	Mismatch Repair
MRN	Mre11-Rad50-Nbs1
mRNP	messenger ribonucleoprotein
ncBAF	Non-canonical BAF
NHEJ	Non-Homologous End-Joining
PAF-1	RNAPII-Associated Factor 1
PCNA	Proliferating cell nuclear antigen
PRC	Polycomb Repressor Complex
PTMs	Post-translational modifications
RBPs	RNA-Binding Proteins
rDNA	ribosomal DNA
REZ	R-loop Elongation Zones
RFSs	Rare Fragile Sites
RIZ	R-loop Initiation Zones
RLFS	R-loop Forming Sequence
RNAPII	RNA polymerase II
RNF168	Ring Finger protein 168
rNMPs	ribonucleotide monophosphates
R-T	Replication-Transcription
SETX	Senataxin
SWI/SNF	SWItch/Sucrose Non-Fermentable
TA-HRR	Transcription-Associated Homologous Recombination Repair
TAM	Transcription-associated mutagenesis
TAR	Transcription-associated recombination
TC-NER	Transcription-Coupled Nucleotide Excision Repair
TDRD3	Tudor domain-containing protein 3 complex
TERRA	Telomeric-Repeat-containing RNA
TGFβ	Transforming growth factor beta
TonEBP	Tonicity-responsive enhancer binding protein
TOP1	DNA topoisomerase 1
TOP2A	DNA topoisomerase 2-alpha
TOP2B	DNA topoisomerase 2-beta
TOP3B	DNA topoisomerase 3 Beta
UFBs	Ultra-fine anaphase bridges
XPG	Xeroderma Pigmentosum group G
Xrn2	5′-3′ exoribonuclease 2
